# Comparative Transcriptome Analysis of Fungal Pathogen *Bipolaris maydis* to Understand Pathogenicity Behavior on Resistant and Susceptible Non-CMS Maize Genotypes

**DOI:** 10.3389/fmicb.2022.837056

**Published:** 2022-04-29

**Authors:** Shweta Meshram, Robin Gogoi, Bishnu Maya Bashyal, Aundy Kumar, Pranab Kumar Mandal, Firoz Hossain

**Affiliations:** ^1^Division of Plant Pathology, Indian Council of Agricultural Research (ICAR)-Indian Agricultural Research Institute, New Delhi, India; ^2^Indian Council of Agricultural Research (ICAR)-National Institute for Plant Biotechnology, New Delhi, India; ^3^Division of Genetics, Indian Council of Agricultural Research (ICAR)-Indian Agricultural Research Institute, New Delhi, India

**Keywords:** *Bipolaris maydis* race “O”, non-CMS maize, RNA-seq, host–pathogen interaction, differentially expressed genes (DEGs), effectors

## Abstract

*Bipolaris maydis* is pathogen of maize which causes maydis leaf blight disease. In India major losses occur due to the *B. maydis* race “O” pathogen, whereas in other parts of the world, major losses are due to the race “T” pathogen. In the present study, we conducted an *in planta* transcriptomics study of the *B. maydis* race “O” pathogen after infection on non-CMS maize resistant and susceptible genotypes by mRNA sequencing to understand the molecular basis of pathogenicity for better management of the pathogen. Approximately 23.4 GB of mRNA-seq data of *B. maydis* were obtained from both resistant and susceptible maize backgrounds for fungus. Differentially expressed genes (DEGs) analysis of *B. maydis* in two different genetic backgrounds suggested that the majority of highly DEGs were associated with mitochondrial, cell wall and chitin synthesis, sugar metabolism, peroxidase activity, mitogen-activated protein kinase (MAPK) activity, and shikimate dehydrogenase. KEGG analysis showed that the biosynthetic pathways for secondary metabolism, antibiotics, and carbon metabolism of fungus were highly enriched, respectively, in susceptible backgrounds during infection. Previous studies in other host pathogen systems suggest that these genes play a vital role in causing disease in their host plants. Our study is probably the first transcriptome study of the *B. maydis* race “O” pathogen and provides in-depth insight of pathogenicity on the host.

## Introduction

*Bipolaris maydis* (*Cochliobolus heterostrophus*) is a necrotrophic ascomycete belonging to the order Pleosporales, which causes maydis leaf blight (MLB) or southern corn leaf blight. In India, yield losses occur due to the *B. maydis* race “O” pathogen in maize, unlike the rest of the world where major losses are due to the race “T” pathogen. Maize (*Zea mays* L.) is the third most widely grown cereal crop after rice and wheat in India (Hussain, 2011). Losses in India due to MLB disease may extend up to 70% (Kumar et al., 2016). Infected maize typically causes tan and elliptical to rectangular lesions (White, 1999) on the leaves and under the surface of foliage which later coalesce and result in an extensive blight appearance. Among the 65 major foliar diseases of maize, MLB is an important disease of maize (Rahul and Singh, 2002). MLB is reported from almost all maize growing regions in the world but more severe in areas where environmental conditions are hot and humid. Three races (C, O, and T) of *B. maydis* have been identified in maize crop so far. Race “O” is more prevalent than “T” in India, whereas worldwide, race “T” is a major concern; race “C” is reported only in China (Mubeen et al., 2017).

*Bipolaris maydis* race “O” is predominant in tropical and sub-tropical areas. It infects a broad range of maize genotypes including CMS and non-CMS maize lines. Studies reported that inoculated susceptible lines with race “O” showed a 50% yield loss (Fisher et al., 1976; Gregory et al., 1978). Typical symptoms of race “O” are small lesions which eventually become diamond-shaped and rectangular as they mature and are restricted to leaf veins (Ali et al., 2011). Race “T” attacks CMS maize which promotes Texas male-sterile cytoplasm (cms-T), this race historically caused an epidemic in the United States in 1970 and 1971. Typical symptoms of race “T” develop on leaves, husks, and ears, and produce small lesions on maize (Ullstrup, 1972). Races “O” and “T” can be identified best with a host differential test, *viz*., a pathogenicity test of cms-T plants, and also by physiological/morphological characteristics on culture media (Leonard, 1977; Warren et al., 1977).

Combatting losses caused by MLB resistance varieties is the best solution. Maize crop is resistant to race “T” with normal cytoplasm therefore management of race “T” can be achieved with elimination of cms-T from cultivars of high agronomic importance (Hyre, 1970; Ullstrup, 1972). In India, a broad range of maize genotypes serve as the major host of race “O,” which causes huge loss. So far, we only know that the rhm recessive gene of *C. heterostrophus* confers resistance to race “O” (Zaitlin et al., 1993). Various screening techniques, *viz*., detached leaf techniques (Lakshmi and Sharma, 1987), tissue culture (Kuehnle and Earle, 1988), and seedling assays (Tajimi et al., 1985) have been investigated for disease resistance. Conventional breeding or recurrent selection is also an effective method to improve resistance against MLB (Shieh and Lu, 1993). On the pathogen side, very few studies have been conducted to understand the race “O” pathogen. In the present study, whole transcriptome analysis was done by mRNA sequencing of the *B. maydis* race “O” pathogen after infection of resistant and susceptible non-CMS maize inbred lines to understand the molecular basis of pathogenicity leading to better management of the pathogen. The race “O” pathogen used in this study was re-confirmed by Venkatesh et al. (2021). So far transcriptome analysis of fungal pathogen *B. sorokiniana* on infected wheat (Ye et al., 2019) and *B. sorghicola* on sorghum (Mizuno et al., 2012; Yazawa et al., 2013) has been completed. Here we present probably the first *in planta* transcriptome study of the *B. maydis* race “O” pathogen on non-CMS maize lines. RNA-seq and fold change were calculated by comparing *B. maydis* infection on susceptible inoculated (SI) versus resistant inoculated (RI) lines.

## Materials and Methods

### Plant Material and Fungal Inoculation

**FIGURE 1:**
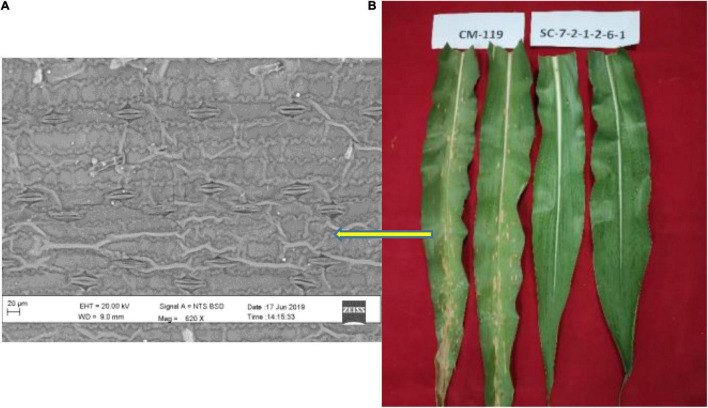
**(A)** Scanning electron micrograph showing abundant fungal hyphae in susceptible (CM 119) line after infection with *Bipolaris maydis*. **(B)** Symptoms of maydis leaf blight on susceptible CM 119 (score 4) and resistant SC-7 (score 1) and both genotypes under field conditions.

Two extreme genotypes of maize inbred lines differing in their susceptibility to *B. maydis* were used in this study. Line SC-7-2-1-2-6-1 (SC-7) which is registered (INGR 07025) as a highly resistant non-CMS line and CM 119 which is established as a standard susceptible marker against *B. maydis*. The experiment was conducted under greenhouse conditions. The *B. maydis* New Delhi isolate was maintained in pure culture and later mass-multiplied on soaked sorghum seeds. After 30 days, old plants were inoculated with pathogen *B. maydis* according to the method described by Payak and Sharma (1983). Inoculated samples were collected for RNA-seq at 48 h post inoculation (disease phase, Liu et al., 2015). Symptoms started appearing and fungal signs were noticed more clearly on susceptible line CM 119 (Figure 1).

### RNA Extraction, Library Preparation, and Sequencing

Total RNA was extracted from infected CM 119 and SC-7 at 48 h post inoculation and their non-inoculated controls with an RNAeasy plant mini kit (Qiagen) following the manufacturer’s instructions. Total RNA of each sample was quantified and qualified by an Agilent 2100 Bioanalyzer (Agilent Technologies, Palo Alto, CA, United States), NanoDrop (Thermo Fisher Scientific Inc.), and 1% agarose gel. One microgram of total RNA with an RIN value above 7 was used for the following library preparation. Next-generation sequencing library preparations were carried out as instructed in the manufacturer’s protocol (NEBNext^®^ Ultra™ RNA Library Prep Kit for Illumina^®^).

### Quality Control and Read Mapping to the Reference Genome

Quality checks for the raw fastq files were conducted through a pipeline consisting of FastQC. The minimum quality score was 30 (Qphred). HISAT2 software was selected according to the characteristics of the reference genome. The reference genome used for this study was Bipolaris_maydis_c5_gca_000338975.CocheC5_3.dna.toplevel.fa. Raw read counts mapped to each gene from the HiSat2-generated alignments were obtained using the feature counts command (Liao et al., 2014) of the subread package (Liao et al., 2013).

### Differential Gene Expression Analysis

For differentially expressed gene (DEG) identification, DESEq2 V1.21.17 with a replicated package was run with parametric fit Padj < 0.05. A false discovery rate (FDR) of 0.05 and a fold change of >0 were set as thresholds for DEG calling, as previously described (Bagnaresi et al., 2012; Li and Lan, 2015) and *P*-value >0.05 was set. The list of all DEGs is provided (Supplementary Table 1) to allow any further DEG sub-setting based on different FDRs or fold changes.

### GO Enrichment and KEGG Analyses

GO enrichment analyses were conducted with topGO, an R-bioconductor package for enrichment analysis version 2.28.0., and a *P*-value of 0.001 was used with classic Fisher ordering, ranks= topgoFisher. The Bioconductor package ClusterProfiler version 3.10.0 was used to generate relevant KEGG pathway pictures incorporating color-coded expression values (Padj < 0.05). A pie chart for GO enrichment is provided along with enriched genes (Supplementary Tables 2–4).

### qRT-PCR for Expression of Selected Genes

**FIGURE 2:**
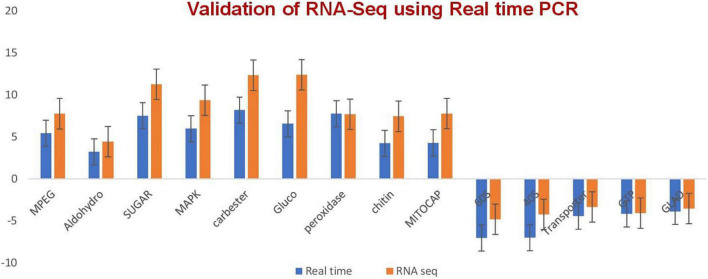
qRT-PCR validation of the relative expression data of genes obtained in RNA-seq analysis. Expression levels of selected transcripts are shown in dark blue (qRT-PCR) and orange (RNA-seq). The fungal actin gene was used for transcript normalization of the signal intensity which is shown on the *y* axis. The *x* axis shows comparisons of the results of the two analyses. Error bars show standard deviations for triplicate assays.

The validation of the RNA-seq technique was performed by quantitative RT-PCR through monitoring the expression levels of seven selected transcripts (Figure 2 and Supplementary Table 5) after designing primers for selected genes (Supplementary Table 6). A melt curve is also provided in Supplementary Figure 1.

### Statistical Analysis

qRT-PCR data were analyzed by analysis of variance (ANOVA) using the Statistical Package for Social Science (SPSS, IBM, Chicago, IL, United States) version 16.0. The statistical significance was judged at *P* < 0.05.

## Results

### Disease Development

There were no observable phenotypic differences between the susceptible and resistant maize inbred lines without pathogen inoculation at all-time points. We observed prominent symptoms on susceptible line CM 119. Non-inoculated controls never showed necrotic lesions. Lesions were visible from 72 h after inoculation. In the resistant line, lesions were small and fewer in numbers. There was a noticeable symptom difference between the susceptible and resistant maize inbred lines at 96 h after inoculation (Figure 1).

### Sequencing and Mapping

From the sequencing of *in planta* libraries, approximately 31,994,420 resistant and 46,087,568 susceptible lines reads were generated (Table 1). The genome was mapped with reference genome Bipolaris_maydis_c5_gca_ 000338975.CocheC5_3.dna.toplevel.fa., and mapping statistics are provided in Supplementary Figure 2.

**TABLE 1:** Summary of the Illumina sequence reads obtained from *Zea mays* plants inoculated with *B. maydis* pathogen grown on sorghum seeds.

Sample*	Total clean reads	Data (GB)	Q30%	Paired total	Paired aligned uniquely	Unpaired total	Unpaired aligned uniquely	Overall alignment rate
RI	34527322	5.24	89.8	17263661	17215458	34430916	56440	0.45
RIR	29461518	4.46	90.54	14730759	14687660	29375320	59682	0.5
SI	42163024	6.3	91.11	21081512	20621885	41243770	68621	2.35
SIR	50012112	7.5	90.23	25006056	23866276	47732552	171279	4.91

*RI, resistant inoculated; RIR, resistant inoculated replicate; SI, susceptible inoculated; SIR, susceptible inoculated replicate.

### Differential Gene Expression Analysis

**FIGURE 3:**
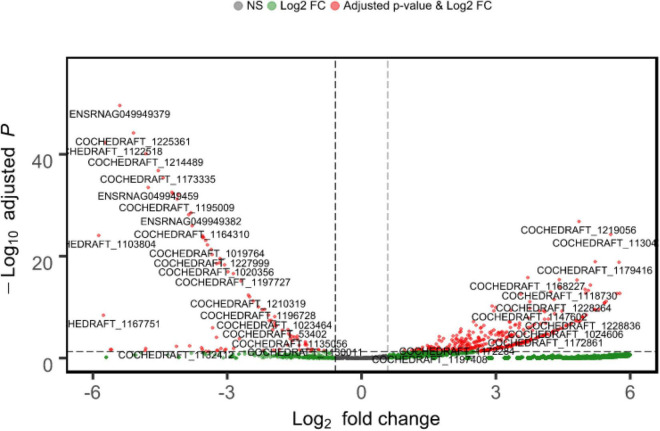
Mean expression versus log fold change plots (MA-plots). Transcriptional changes of *B. maydis* are presented in SC-7 and CM 119 48 h post inoculation. Normalized *P*-values are plotted versus Log2 fold changes. Genes with an FDR < 0.05 are plotted.

Approximately 23.4 GB of mRNA-seq data of *B. maydis* were obtained from both resistant and susceptible maize backgrounds. DEG analysis was conducted to detect *B. maydis* transcriptome changes during the pathogenesis of maize. Out of 10,363 mapped genes, 3313 genes were upregulated and only 100 genes were downregulated, and 6949 genes were commonly expressed in *B. maydis* SI plants compared to RI plants. A list of genes and their expression is provided in Supplementary Table 1. Log FCs and *P*-value are shown in Figure 3.

Transcriptome analysis of the top 40 DEGs suggested 20 upregulated and 20 downregulated genes, the detailed description of genes along with their fold change and function is provided in Tables 2, 3. Further, these DEGs were studied for genes related to pathogenesis and pathogen fitness which revealed important aspects, as shown in Table 4 and Supplementary Table 1. On the other hand, the data were also analyzed *in silico* to find out putative effectors using the Effector database and 22 effectors were predicted (Table 5).

**TABLE 2:** Top 20 highly upregulated genes of *B. maydis* differentially expressed in inoculated resistant and susceptible plants.

Gene ID	Log fold	Annotation	Protein ID
COCHEDRAFT_1021363	12.60451532	Domain of unknown function (DUF3328)	EMD91267
COCHEDRAFT_1093124	10.37257746	EthD domain	EMD94050
COCHEDRAFT_1202737	12.82138123	Cerato-platanin	EMD92796
COCHEDRAFT_1101487	12.45535783	Domain of unknown function (DUF3328)	EMD91266
COCHEDRAFT_1140189	12.39934756	Glucanosyl transferase	EMD90599
COCHEDRAFT_1140839	12.33128367	Carboxylesterase family	EMD89062
COCHEDRAFT_16026	7.231602644	1941118.1	EMD92794
COCHEDRAFT_1185072	12.98058241	Tannase and feruloyl esterase	EMD86863
COCHEDRAFT_1147694	13.19280605	13684.SNOT_06000	EMD85980
COCHEDRAFT_1198613	7.756361319	MAPEG family	EMD85630
COCHEDRAFT_1028180	6.882906745	Cutinase	EMD94256
COCHEDRAFT_1134453	14.34810723	EXS family	EMD92204
COCHEDRAFT_1193999	14.27393273	Cytochrome P450	EMD92530
COCHEDRAFT_1222232	15.00590358	GMC oxidoreductase	EMD94987
COCHEDRAFT_1166191	6.417942179	Alcohol dehydrogenase GroES-like domain	EMD95758
COCHEDRAFT_1019411	6.282567882	Inherit from ascNOG: conserved hypothetical protein	EMD95841
COCHEDRAFT_1219056	4.855362398	Bi functional enzyme with both catalase and broad spectrum peroxidase activity (by similarity)	EMD85713
COCHEDRAFT_1113526	6.25668138	D-Isomer specific 2-hydroxyacid dehydrogenase, catalytic domain	EMD87549
COCHEDRAFT_1224654	6.163316228	Aldehyde dehydrogenase family	EMD91526
COCHEDRAFT_1130430	5.564541045	Flavodoxin-like fold	EMD94134

**TABLE 3:** Top 20 highly downregulated genes of *B. maydis* differentially expressed in inoculated resistant and susceptible plants.

Gene ID	Log fold	Annotation	Protein ID
COCHEDRAFT_1195	−3.861184569	40s ribosomal protein	EMD89020
COCHEDRAFT_1177804	−4.236671634	40S ribosomal protein S3	EMD89815
COCHEDRAFT_1021505	−4.212183943	60S ribosomal protein	EMD91574
COCHEDRAFT_1225360	−3.861184569	L 21 protein	EMD89740
COCHEDRAFT_1157448	−4.430696422	40S ribosomal protein S3	EMD90435
COCHEDRAFT_1173335	−4.540817315	40S ribosomal protein S1	EMD91948
COCHEDRAFT_1214489	−4.814349769	60s ribosomal protein l11	EMD91114
COCHEDRAFT_1225361	−5.094309904	40S ribosomal protein S9	EMD89741
COCHEDRAFT_1127623	−3.48264224	40S ribosomal protein S8	EMD96126
COCHEDRAFT_1224611	−3.530642646	Ribosomal protein	EMD91465
COCHEDRAFT_1145202	−3.550348853	Glyceraldehyde-3-phosphate dehydrogenase	EMD87494
COCHEDRAFT_1019764	−3.442593019	Ribosomal protein S13/S18	EMD94767
COCHEDRAFT_1164310	−3.783863683	Ribosomal protein L11, N-terminal domain	EMD97422
COCHEDRAFT_1139445	−4.098487448	Promotes the GTP-dependent binding of aminoacyl-tRNA to the A-site of ribosomes during protein biosynthesis (by similarity)	EMD90278
ENSRNAG049949382	−3.809190151	–	
ENSRNAG049949379	−5.40016	–	
ENSRNAG049949459	−4.76472	–	
COCHEDRAFT_1122518	−5.724881452	H3	EMD84642
COCHEDRAFT_1103804	−5.871461339	3027035.1	EMD91248

**TABLE 4:** Important upregulated genes for pathogen fitness and pathogenesis.

Gene and gene ID	Log fold change	Description	References
**Mitochondrial genes**			
Mitochondrial carrier protein COCHEDRAFT_1139719	7.7685614	Transport of ions, nucleotide, amino acid, and cofactors across membrane	Bertrand, 2000; Arcila et al., 2021
Mitochondrial 18 kDa protein COCHEDRAFT_1024553	8.172178	Mitochondrial fission, morphology, and development of mitochondria, mutation leads to apoptosis	
AMP-binding enzyme COCHEDRAFT_1105220	10.68332	Mitochondrial biogenesis	
ATP-dependent serine protease COCHEDRAFT_1207993	8.95483288	Participates in the regulation of mitochondrial gene expression and in the maintenance of the integrity of the mitochondrial genome	
YmL38 YmL34 7.407734745	7.407734745	Mitochondrial 54S ribosomal protein	
Mitochondrial protein synthesis	7.976810257	Promotes mitochondrial ribosomes in a GTP-dependent manner	
**Fungal cell wall and chitin synthesis genes**			
Chitin synthase III catalytic subunit COCHEDRAFT_1197445	7.451803741	Responsible for the synthesis of the majority of the chitin found in the cell wall periphery	Banks et al., 2005; Langner and Göhre, 2016; Pusztahelyi, 2018; Garcia-Rubio et al., 2020
Chitin synthase. COCHEDRAFT_1192892	10.12960501	Chitin synthases (CHSs) are key enzymes in the biosynthesis of chitin, an important structural component of fungal cell walls	
Chitin synthesis regulation, resistance to Congo red COCHEDRAFT_1152857	3.273618	Regulates chitin deposition in the fungal cell wall	
**Sugar metabolism**			
Sugar transporter COCHEDRAFT_1186319	11.2559271	Sugar transporters (STs) that are essential for taking up the mono- and short oligosaccharides, resulting from extracellular enzymatic digestion of lignocellulose, into the fungal cell	Vankuyk et al., 2004; Lv et al., 2020; Monfared et al., 2020
Glucanases COCHEDRAFT_1118047	7.355847304	Plays a role in cell expansion during growth, in cell–cell fusion during mating, and in spore release during sporulation. This enzyme may be involved in beta-glucan degradation	
**Genes related to toxin (polyketide cyclases)**			
Polyketide cyclases family COCHEDRAFT_1167946	7.332835	T-toxin is a family of linear polyketides 37–45 carbons in length, of which the major component is 41 carbons	Gaffoor et al., 2005; Schindler and Nowrousian, 2014
Acetoacetate decarboxylase COCHEDRAFT_1103773	8.894696	Catalyzes the conversion of acetoacetate to acetone and carbon dioxide	
LAM1 COCHEDRAFT_1167244	8.228607	3-Hydroxyacyl-CoA dehydrogenase, NAD-binding domain	
**Other important genes associated with secondary metabolites and signaling**			
Peroxidase COCHEDRAFT_1125365	7.721354	Peroxidases are a group of oxidoreductases which mediate electron transfer from hydrogen peroxide (H_2_O_2_) and organic peroxide to various electron acceptors	Mir et al., 2015
Reactive mitochondrial oxygen species modulator COCHEDRAFT_1022035	8.233186	Required in fungal differentiation processes that are necessary for virulence	Hansberg et al., 2012; Mir et al., 2015; Martínez-Soto and Ruiz-Herrera, 2017
Mitogen-activated protein kinase COCHEDRAFT_1207640	9.375412764	Involved in fungal development, sexual reproduction, pathogenicity and/or virulence in many filamentous plant pathogenic fungi	
Catalase COCHEDRAFT_1179052	8.872981819	Catalyzes the reaction of cyanate with bicarbonate to produce ammonia and carbon dioxide	
Shikimate dehydrogenase substrate binding domain COCHEDRAFT_1228346	9.422273	Catalytic domain at N terminus binds to the substrate, 3-dehydroshikimate	
Signal-recognition-particle (SRP) COCHEDRAFT_1187286	8.077532	Signal-recognition-particle assembly has a crucial role in targeting secretory proteins to the rough endoplasmic reticulum membrane	

**TABLE 5:** List of candidate effector genes identified in *B. maydis*.

Gene ID	Annotation	CDS	Effector prediction	Description based on UniProt/InterPro and similarity
COCHEDRAFT_1093124	EthD domain Ethyl tert-butyl ether degradation	103	0.916	Contributes to conidial pigmentation that provides protection from UV radiation, heat and cold stress
COCHEDRAFT_1202737	Hypothetical protein	138	0.918	Protein occurs in the cell wall of the fungus and is involved in the host-plane interaction and induces both cell necrosis and phytoalexin synthesis which is one of the first plant defense-related events
COCHEDRAFT_1134423	Hypothetical protein	130	0.999	Component of the endoplasmic reticulum-associated degradation (ERAD) pathway
COCHEDRAFT_1155213	Hypothetical protein	162	0.997	Integral component of cell membrane
COCHEDRAFT_1193149	Conserved hypothetical protein	165	0.691	Integral component of cell membrane
COCHEDRAFT_1020438	Conserved hypothetical protein	86	0.539	NADH dehydrogenase (ubiquinone) 1 alpha subcomplex subunit 1
COCHEDRAFT_1094426	Conserved hypothetical protein	211	0.888	Thioredoxin-like fold domain-containing protein
COCHEDRAFT_1168141	Hypothetical protein	169	0.965	Uncharacterized protein
COCHEDRAFT_1149474	Proteasome subunit	205	0.767	Cleavage of peptide bonds with very broad specificity, endopeptidase
COCHEDRAFT_1199047	ATP synthase E chain	90	0.71	Mitochondrial membrane ATP synthase
COCHEDRAFT_1024634	Cytidine and deoxycytidylate deaminase zinc-binding region	185	0.948	Scavenges exogenous and endogenous cytidine and 2′-deoxycytidine for UMP synthesis
COCHEDRAFT_1188666	Antibiotic biosynthesis monooxygenase	108	0.992	ABM domain-containing protein
COCHEDRAFT_1148964	Hypothetical protein	72	0.966	Membrane-associated and mitochondrion-associated cellular component
COCHEDRAFT_1207896	Synaptobrevin	200	0.976	Vesicle-mediated transport
COCHEDRAFT_1088730	Dienelactone hydrolase family	250	0.555	DLH domain, hydrolase activity
COCHEDRAFT_1019519	Redoxin	167	0.856	Thiol-specific peroxidase that catalyzes the reduction of hydrogen peroxide and organic hydroperoxides to water and alcohols, respectively. Plays a role in cell protection against oxidative stress by detoxifying peroxides
COCHEDRAFT_1221715	Protein of unknown function (DUF1687)	150	0.98	Putative mitochondrial redox protein which could be involved in the reduction of small toxic molecules
COCHEDRAFT_1221723	Ubiquitin-conjugating enzyme	149	0.779	Glycyl thioester intermediate, ATP binding, transferase activity
COCHEDRAFT_1019537	Mitochondrial ribosomal protein L51/S25/CI-B8 domain	93	0.986	Electron transport, respiratory chain
COCHEDRAFT_1166992	ATP synthase delta (OSCP) subunit	231	0.978	ATP synthase subunit 5, mitochondrial, proton-transporting ATP synthase activity
COCHEDRAFT_1191234	Cytochrome *c* domain-containing protein	108	0.999	Electron carrier protein. The oxidized form of the cytochrome *c* heme group can accept an electron from the heme group of the cytochrome c1 subunit of cytochrome reductase. Cytochrome *c* then transfers this electron to the cytochrome oxidase complex, the final protein carrier in the mitochondrial electron-transport chain (by similarity)
COCHEDRAFT_1127633	Stress-response A/B barrel domain-containing protein	110	0.931	Stress-response A/B barrel domain-containing protein

### GO Categories and Enrichment Analysis

**FIGURE 4:**
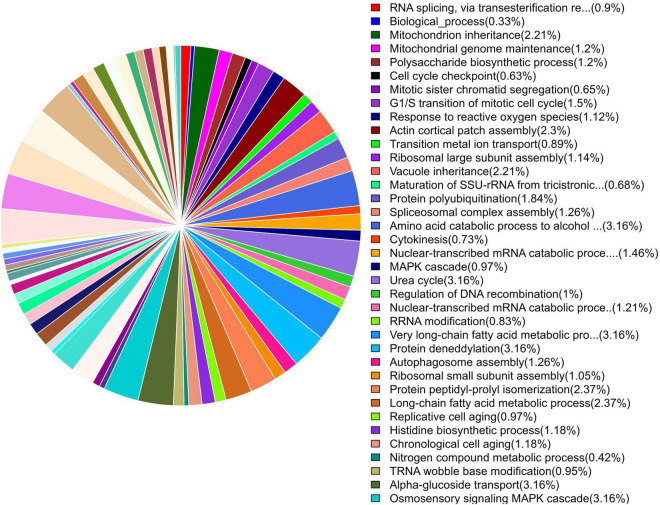
Pie chart of enriched GO terms of *B. maydis* for biological functions differentially expressed in inoculated resistant and susceptible plants.

**FIGURE 5:**
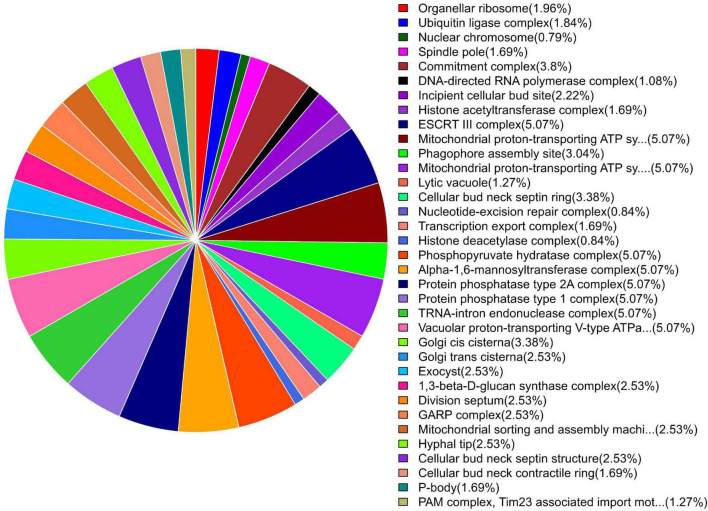
Pie chart of enriched GO terms of *B. maydis* for cellular functions differentially expressed in inoculated resistant and susceptible plants.

**FIGURE 6:**
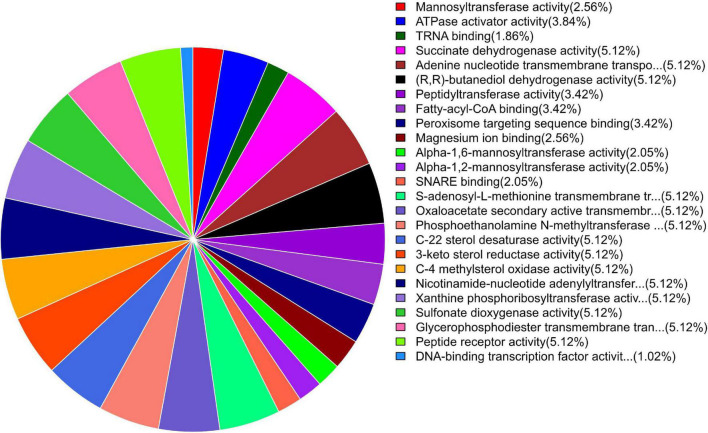
Pie chart of enriched GO terms of *B. maydis* for molecular functions differentially expressed in inoculated resistant and susceptible plants.

GO enrichment suggested that most of the genes under DEGs were associated with pathogen fitness and reproduction. The results are presented in Figures 4–6 and described in Tables 6–8, with additional information available in Supplementary Tables 2–4.

**TABLE 6:** Top 10 important enriched GO terms of *B. maydis* for biological functions differentially expressed in inoculated resistant and susceptible plants.

Gene ID	Annotation	Enriched genes	Function
1	GO:0000003	Reproduction	188
2	GO:0002181	Cytoplasmic translation	67
3	GO:0007275	Multicellular organism development	41
4	GO:0000054	Ribosomal subunit export from nucleus	21
5	GO:0000096	Sulfur amino acid metabolic process	21
6	GO:0000122	Negative regulation of transcription	22
7	GO:0000272	Polysaccharide catabolic process	21
8	GO:0000375	RNA splicing *via* transesterification	33
9	GO:0008150	Biological process	730
10	GO:0000001	Mitochondrion inheritance	14
			

**TABLE 7:** Top 10 important enriched GO terms of *B. maydis* for cellular functions differentially expressed in inoculated resistant and susceptible plants.

Gene ID	Annotation	Enriched genes	Number
1	GO:0005575	Cellular component	555
2	GO:0000139	Golgi membrane	72
3	GO:0000322	Storage vacuole	58
4	GO:0000502	Proteasome complex	25
5	GO:0000313	Organellar ribosome	22
6	GO:0000151	Ubiquitin ligase complex	12
7	GO:0000228	Nuclear chromosome	15
8	GO:0000922	Spindle pole	10
9	GO:0000243	Commitment complex	6
10	GO:0000428	DNA-directed RNA polymerase complex	10

**TABLE 8:** Top 10 important enriched GO terms of *B. maydis* for molecular functions differentially expressed in inoculated resistant and susceptible plants.

Gene ID	Annotation	Enriched genes	Number
1	GO:0003674	Molecular function	626
2	GO:0000166	Nucleotide binding	97
3	GO:0000030	Mannosyl transferase activity	10
4	GO:0001671	ATPase activator activity	3
5	GO:0000049	tRNA binding	4
6	GO:0000104	Succinate dehydrogenase activity	2
7	GO:0000295	Adenine nucleotide transmembrane transport	2
8	GO:0000721	(R,R)-butanediol dehydrogenase activity	2
9	GO:0000048	Peptidyltransferase activity	2
10	GO:0000062	Fatty-acyl-CoA binding	2
			

## Discussion

**FIGURE 7:**
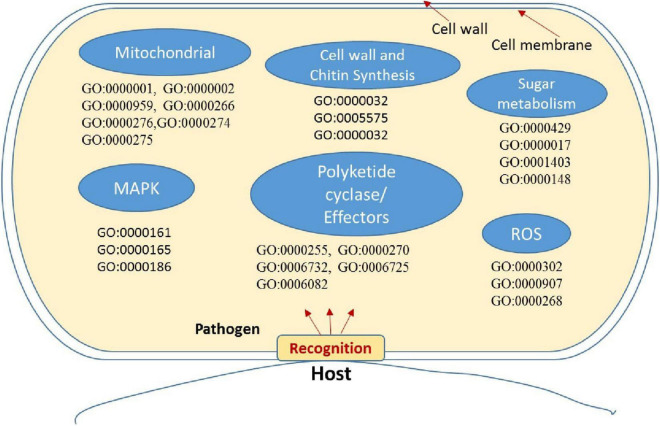
Putative representation of possible activation of genes and GO enriched terms in fungal pathogen *Bipolaris maydis* in susceptible maize (CM 119) compared to resistant maize (SC-7) during early stages of infection. GO term and associated gene IDs are provided in Supplementary Table 7.

**FIGURE 8:**
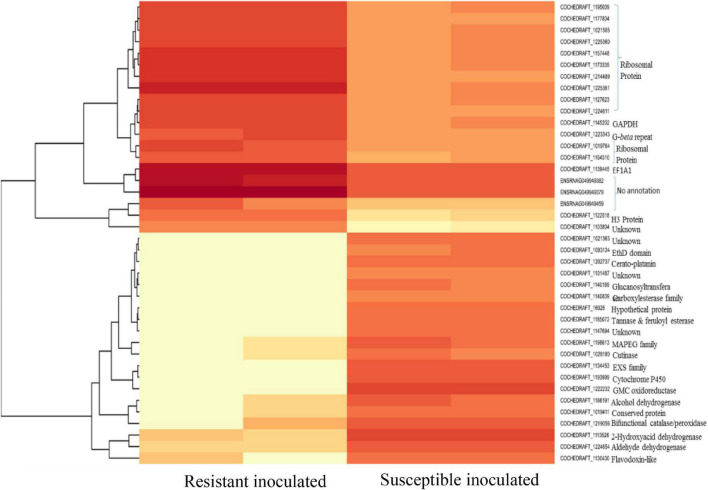
Heat map of top 40 differentially expressed genes of *B. maydis* in resistant and susceptible maize genotypes 48 h post inoculation.

**FIGURE 9:**
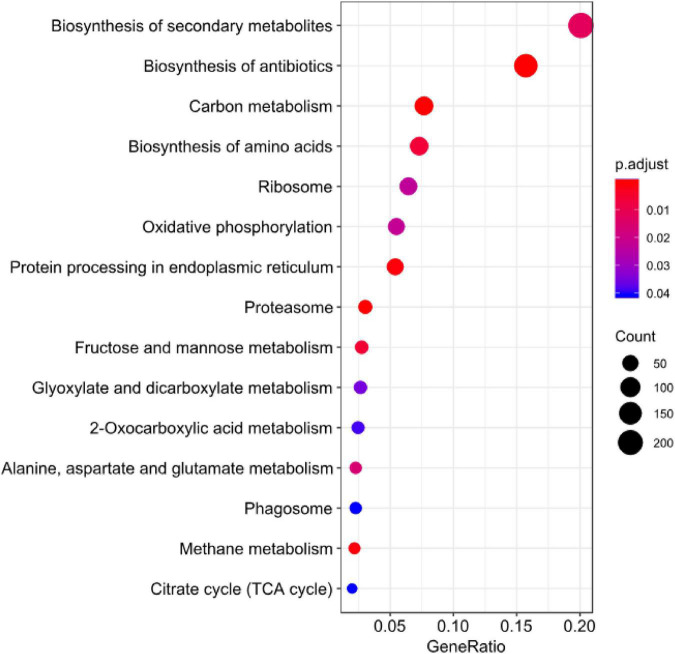
KEGG enrichment analysis for DEGs of *B. maydis* in maize susceptible and resistant genotypes at 96 h post inoculation.

Enrichment analysis and the expression patterns of the highly upregulated genes indicated that successful pathogenicity of *B. maydis* depends on pathogen fitness genes such as mitochondrial genes, cell wall synthesis, toxin-related effector molecules, and on other hand, cell wall degradation of the host, detoxification, and host defense evasion (Figures 7–9). Understanding the pathogen’s molecular pathways during the infection process using transcriptome analysis can contribute significantly to identifying new targets for SSR control, novel genes, and pathogenicity-related pathways (Ribeiro et al., 2020; Poretti et al., 2021).

### Mitochondrial Genes

Mitochondria have diverse functions to perform in fungal cells. Mitochondria play a major role in fungal metabolism and fungicide resistance (Arcila et al., 2021). In the present study, genes associated with mitochondrial functions for *B. maydis* race “O” were upregulated in the susceptible line (CM 119), which suggests that CM 119 supports the growth of *B. maydis*, and on the other hand, SC-7 restricts the cellular activity of fungus (Figure 8). Previously studies suggested that fungal mitochondria play a significant role in determining fungal fitness and virulence (Calderone et al., 2015; Medina et al., 2020). A recent study suggests that endoplasmic reticulum (ER) and mitochondrial interactions along with the ER-mitochondria organizing network (ERMIONE) play important roles in adaptive responses in fungi, particularly in response to cell surface-related mechanisms that facilitate fungal invasion, growth, and stress responsive behaviors that support fungal pathogenicity (Koch et al., 2017). An investigation on *C. parasitica* study suggested the role of mitochondria in hypo virulence (Bertrand, 2000). Another study on the mitochondria genome of phytopathogens *Synchytrium endobioticum* and *Phlebia radiate* showed that alteration in the mitochondrial genome majorly affects the ability of fungi to adapt to changing environments (Medina et al., 2020). Overall, fungal mitochondria play a crucial role in determining pathogenicity of the host, and in susceptible backgrounds of the host, these genes are more expressed whereas resistant genotypes have a tendency to suppress the mitochondrial genes and ultimately the pathogen becomes less virulent, which we found in the present study.

### Fungal Cell Wall and Chitin Synthesis Genes

The cell wall is an important component of fungal cells which mediates fungal cell interactions with its external environment and hyphal development (Castro et al., 2022). Chitin is the main component for cell wall synthesis. The cell wall protects the cell content, provides rigidity, and determines the cellular structure. It also protects the cell from various stresses including osmotic changes which are significant for healthy fungal cells. It also carries some proteins that play a role in recognition, adhesion, and receptor activity. There are several studies which establish the role of the fungal cell wall in pathogen fitness and its association with pathogenesis (Banks et al., 2005; Langner and Göhre, 2016; Pusztahelyi, 2018; Garcia-Rubio et al., 2020). It has also been investigated whether the fungal cell wall plays a crucial role in spore development (Backes et al., 2020) and in antifungal resistance activity (Díaz-Jiménez et al., 2012). In the present study, the high expression of cell wall-associated genes reconfirmed the fact that the fungal cell wall plays an essential role in disease development in susceptible hosts and also showed that the resistant genotype had the capacity to hinder the expression of fungal cell wall genes during the interaction (Figure 7).

### Sugar Metabolism

Sugar transporter genes of filamentous fungi are associated with multiple physiological and biochemical processes, such as the response to various stresses (Vankuyk et al., 2004; Lv et al., 2020; Monfared et al., 2020). They were also found to be linked with many salt tolerance and sophisticated transcriptional processes. In the present transcriptome profile of *B. maydis*, genes of sugar metabolism (Figure 9) such as sugar transporter and glucanases were upregulated which suggests that these genes play an essential role in pathogen proliferation under susceptible backgrounds of the host.

### Gene-Related Polyketides

Polyketides (PKs) play a role in mycelial growth and development of asexual and sexual structures of fungi (Gaffoor et al., 2005; Schindler and Nowrousian, 2014) including shikimate dehydrogenase (Kinghorn, 2000). A study on *B. maydis* race “T” demonstrated the association of a toxin-related locus with polyketide biosynthesis and high virulence on T-cytoplasm maize (Rose et al., 2002), similarly in the present study, high expression of PKs was investigated which suggests there could be a possible association of toxin “O” with PKs.

### Other Important Genes Associated With Secondary Metabolites and Signaling

Recent studies proposed peroxidases, catalases, and reactive oxygen species (ROS) as components of the antioxidant defense system in fungal pathogens and were also associated with conidial production (Zhang et al., 2020). A study on fungal pathogen *Magnaporthe oryzae* suggested significant and positive correlations among sensitivity to H_2_O_2_, peroxidase activity, and fungal pathogenicity (Garre et al., 1998; Hansberg et al., 2012; Mir et al., 2015; Chittem et al., 2020). On the other hand, mitogen-activated protein kinase (MAPK) signaling pathways play an important role in cell cycle control, mating, morphogenesis, response to different stresses, resistance to UV radiation, temperature changes, cell wall assembly and integrity, degradation of cellular organelles, virulence, cell–cell signaling, fungus-plant interaction, and response to damage-associated molecular patterns (DAMPs) (Martínez-Soto and Ruiz-Herrera, 2017). Upregulation of these genes in the *B. myadis* race “O” pathogen indicated the interconnected nature in determining the fungal infection strategy in susceptible hosts (Figure 7).

### Candidate Effector Genes Identified in *Bipolaris maydis*

At least 22 transcripts showing homology to genes previously reported to be involved in fungal infection were predicted as effector proteins (Table 5 and Figure 9) in the Effector database using the CSIRO tool EffectorP2 (a machine learning method for fungal effector prediction in secretomes) (Sperschneider et al., 2018). The majority of them showed a probability above 60–90%. This fact can provide evidence for the pathogenicity behavior of *B. myadis* race “O” on susceptible lines.

## Conclusion

Based on the observation of present and previous studies in other host pathogen systems, we suggest that the above cited genes play a vital role in causing disease in their host plants. The DEG study of pathogen genes can provide evidence for its sensitive targets, virulence toward hosts, and resistance against chemicals. This is probably the first transcriptome study of the *B. maydis* pathogen during infection in a non-CMS maize genotype, differing in their susceptibility to the pathogen. The findings from this study emphasize the role of mitochondrial-associated genes and pathways. In addition, cell wall synthesis, genes related to synthesis of polyketides, toxins, and putative candidate effector genes were found to be the key compounds underlying the pathogenesis of the *B. maydis* race “O” pathogen.

## Data Availability Statement

The datasets presented in this study can be found in online repositories. The names of the repository/repositories and accession number(s) can be found below: NCBI BioProject – PRJNA689117.

## Author Contributions

SM, RG, BB, PM, FH, and AK were involved in the conceptualization of the project, study design, critical inputs, and finalization of the manuscript. PM, AK, and BB were involved in wet lab experiments. BB and PM were involved in bio-informatics analyses and data compilation. SM, RG, BB, and PM drafted the manuscript. All authors have read and approved the final manuscript.

## Conflict of Interest

The authors declare that the research was conducted in the absence of any commercial or financial relationships that could be construed as a potential conflict of interest.

## Publisher’s Note

All claims expressed in this article are solely those of the authors and do not necessarily represent those of their affiliated organizations, or those of the publisher, the editors and the reviewers. Any product that may be evaluated in this article, or claim that may be made by its manufacturer, is not guaranteed or endorsed by the publisher.
