# Supplementary material for: Comparative Transcriptome Analysis of Fungal Pathogen Bipolaris maydis to Understand Pathogenicity Behavior on Resistant and Susceptible Non-CMS Maize Genotypes
Source: Front Microbiol. 2022 Apr 29;13:837056. doi: 10.3389/fmicb.2022.837056 (PMC9100685; doi:10.3389/fmicb.2022.837056)
Supplement: Supplementary file 1 [file Data_Sheet_1.docx]

**
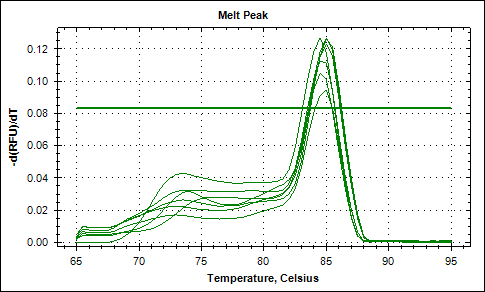

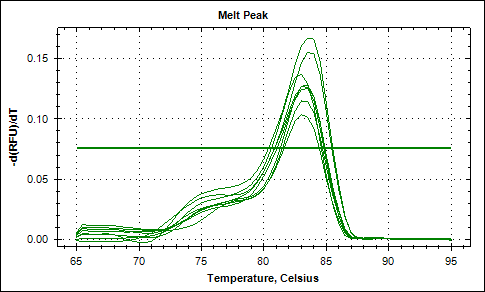
Supplementary Figure S1**: Representative Melt curves of Primers used for validation of RNA Seq data through RT-qPCR

1A. MPEG 1B. Aldohydrolase


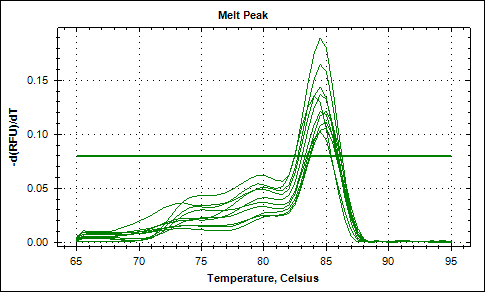

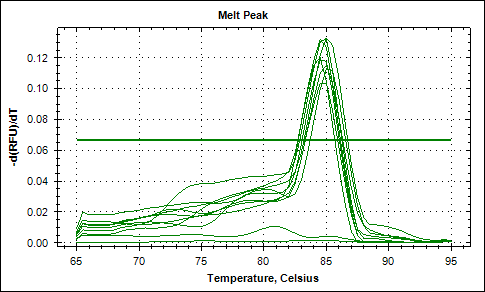


1C. SUGAR 1D. MAPK


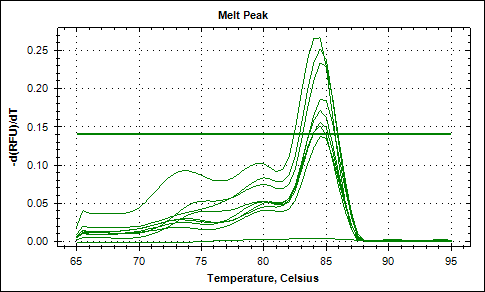

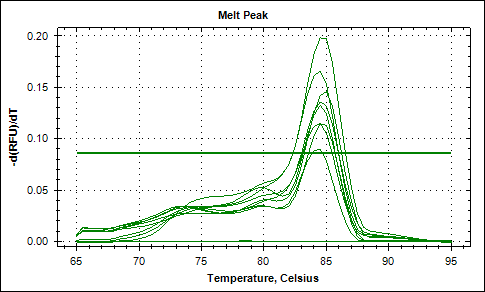


1E. Carbester 1F. Glucanosyl


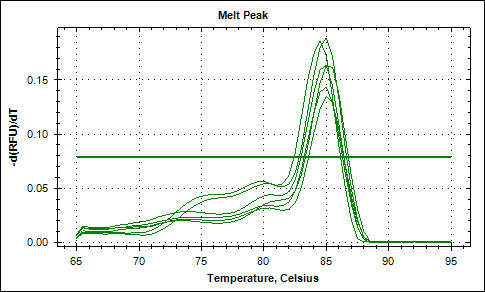

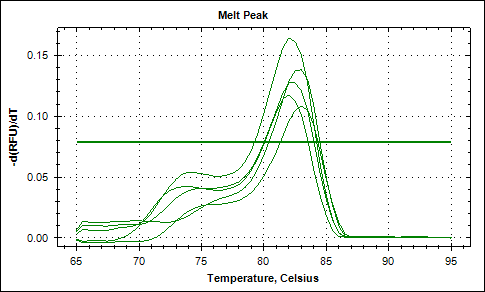


1G. Peroxidase 1H. Chitin


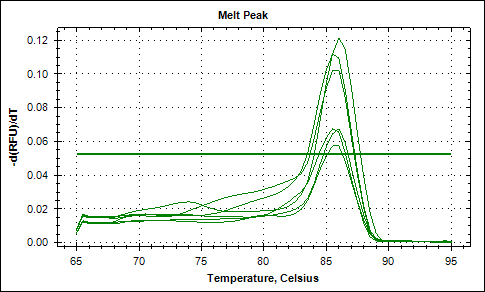

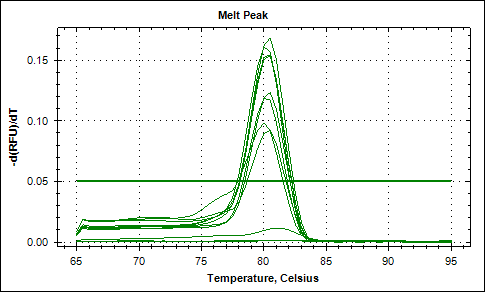


1I. MITOCAP 1J. 60S


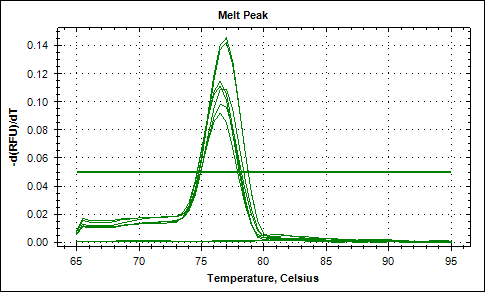

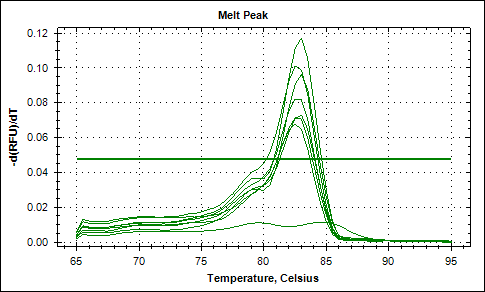


1K. 40S 1L. Transporter


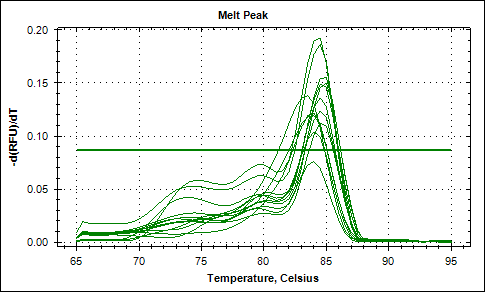

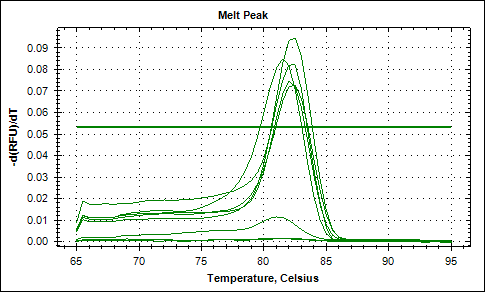


1M. GTP 1N. GLAD
