# Supplementary material for: Comparative Transcriptome Analysis of Fungal Pathogen Bipolaris maydis to Understand Pathogenicity Behavior on Resistant and Susceptible Non-CMS Maize Genotypes
Source: Front Microbiol. 2022 Apr 29;13:837056. doi: 10.3389/fmicb.2022.837056 (PMC9100685; doi:10.3389/fmicb.2022.837056)
Supplement: Supplementary file 7 [file Table_6.docx]

**Supplementary Table S6. Primers designed and validated for fungus using RT PCR**

| **S. no.** | **Gene** | **Sequence** | **Product** | **GC** |
| --- | --- | --- | --- | --- |
| 1 | 40S ribosome F | CTGGACGATAGCACTGGACA | 213 | 59.50 |
|  | R | CTTGTCTGGTTCGGTGTGTG |  |  |
| 2 | Glucanosyl F | GCATCTACCTGGCTCTCGAC | 194 | 59 |
|  | R | AGGAGCGCAGTCAGTCGTAT |  |  |
| 3 | MAPEG F | TCGCACTACTTGGACTCACG | 200 | 59.50 |
|  | R | GCCTTTCCTGTCTCTGATGG |  |  |
| 4 | Carbester F | CGTCGCTCAACAAGTCTCAG | 220 | 58 |
|  | R | GGCAATTTGTGGGCAGTAGT |  |  |
| 5 | MitoCP F | CGCATTTTAGAGGTCCGGTA | 227 | 57 |
|  | R | CGAGGGTGGATTGAAGACAT |  |  |
| 6 | Chitinsynthase F | CGTCGTCTTCTTCCTTCTCG | 191 | 58 |
|  | R | ATGTGGACGCTGATGACGTA |  |  |
| 7 | Sugar F | CGGAGTCGTCAAGACAGTCA | 166 | 59.50 |
|  | R | GTCACCAGACGGGTTACGTT |  |  |
| 8 | Peroxidase F | ACCCAGTCGTGCTTCCTATG | 165 | 59 |
|  | R | GTCTGGCAGGTATTCGTGGT |  |  |
| 9 | MAPK F | TTGGAACCTCTCATCGTTCC | 220 | 60 |
|  | R | GGAAACCAGCAGATCAGAGC |  |  |
| 10 | GLAD F | ACCGAGGACGACATTGTTTC | 208 | 59 |
|  | R | CCTTGCTCTGTCCTGACTCC |  |  |
| 11 | GTP F | AGGTCATCGTCCTCAACCAC | 197 | 58 |
|  | R | CTTGGAGGGAACCATCTTGA |  |  |
| 12 | H3 F | TCGCATCGCAGCATACTAAC | 209 | 62 |
|  | R | TTGTAGCGGTGAGGCTTCTT |  |  |
| 13 | 60S ribosome F | CAAGTACTGCCTCCACGTCA | 230 | 59.50 |
|  | R | GCCTCCCTCTGCTTCTTCTT |  |  |
| 14 | Transporter F | GGTTTCCTTGAGGGTGTCAA | 181 | 60 |
|  | R | AGACGTTGACAAGGGTGGAC |  |  |
| 15 | Aldehydro F | TCGCCCTTTACACCCTACAC | 157 | 59.50 |
|  | R | CGATAGAGCCGGTAAAGCTG |  |  |
