# Supplementary figures and images for: Comparative Transcriptome Analysis of Fungal Pathogen Bipolaris maydis to Understand Pathogenicity Behavior on Resistant and Susceptible Non-CMS Maize Genotypes
Source: Front Microbiol. 2022 Apr 29;13:837056. doi: 10.3389/fmicb.2022.837056 (PMC9100685; doi:10.3389/fmicb.2022.837056)

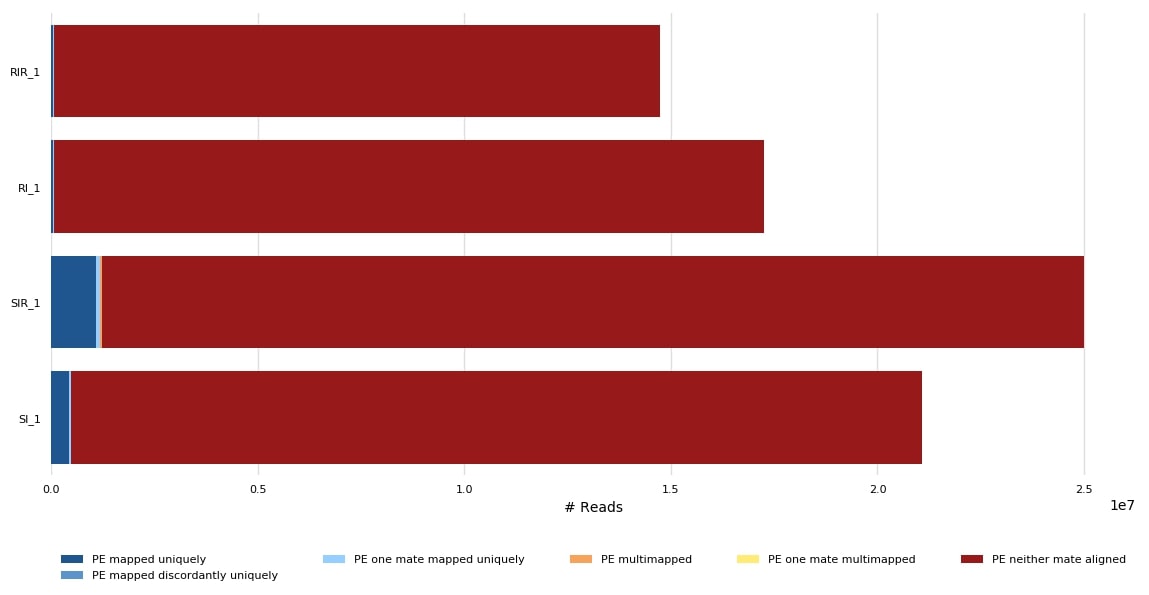

Supplement: Supplementary file 9 [file Image_1.jpg]
